# Transient Type 1 Brugada ECG Pattern in a Patient With Severe Coronary Artery Disease

**DOI:** 10.7759/cureus.117717

**Published:** 2026-10-10

**Authors:** Amaan Khan, Montasir Elmobark, Xiaomei Chen, Neil Swanson, Moaz Ahmad

**Affiliations:** 1 Internal Medicine/Cardiology, James Cook University Hospital, Middlesbrough, GBR; 2 Acute Medicine, University Hospital of North Tees, Stockton-on-Tees, GBR; 3 Medicine, The Rotherham NHS Foundation Trust, Sheffield, GBR; 4 Cardiology, James Cook University Hospital, Middlesbrough, GBR; 5 Cardiology, The Rotherham NHS Foundation Trust, Rotherham, GBR

**Keywords:** brugada electrocardiogram pattern, brugada phenocopy, coronary artery angiography, ecg interpretation, multivessel coronary artery disease (mvcad)

## Abstract

A type 1 Brugada electrocardiographic (ECG) pattern is characteristic of Brugada syndrome but may also occur in association with reversible clinical conditions, including myocardial ischaemia. We describe a 49-year-old man with type 2 diabetes mellitus, severe hypercholesterolaemia and a strong family history of premature ischaemic heart disease who presented with crescendo angina. His admission ECG demonstrated a type 1 Brugada pattern, with subsequent ECGs showing dynamic anterior ischaemic changes. Coronary angiography demonstrated extensive multivessel coronary artery disease, and the patient underwent urgent surgical revascularisation following Heart Team discussion. Serial postoperative ECGs demonstrated complete resolution of the type 1 pattern despite continuation of his regular medication, including mirtazapine. In the absence of arrhythmic syncope, documented ventricular arrhythmia, previous cardiac arrest or a family history of sudden cardiac death, sodium-channel blocker provocation testing was not pursued. Although latent Brugada syndrome cannot be definitively excluded, the temporal relationship between myocardial ischaemia, revascularisation and ECG normalisation supports an ischaemia-associated Brugada phenotype.

## Introduction

Brugada syndrome is an inherited cardiac channelopathy associated with ventricular arrhythmias and sudden cardiac death. The diagnosis has important implications for the patient and their family.

A type 1 Brugada ECG pattern, however, may also occur in other clinical settings, including myocardial ischaemia, electrolyte disturbance, fever and exposure to certain medications. These cases can present a diagnostic challenge, particularly when the ECG abnormality occurs during an acute coronary syndrome. The term Brugada phenocopy has been used to describe Brugada-like ECG patterns arising from an identifiable underlying condition in patients without true congenital Brugada syndrome. Distinguishing these entities is clinically important, as a diagnosis of Brugada syndrome may have implications for risk stratification, family screening and long-term management [1].

We describe a patient with a type 1 Brugada ECG pattern in the setting of extensive coronary artery disease, with complete resolution of the ECG abnormality following surgical revascularisation.

## Case presentation

A 49-year-old man presented to a tertiary centre hospital in the UK with several days of worsening central chest pain occurring both at rest and on exertion, consistent with crescendo angina. His medical history included type 2 diabetes mellitus and severe hypercholesterolaemia, with a total cholesterol of 10 mmol/L. He had a strong family history of premature ischaemic heart disease but no family history of sudden cardiac death. There was no previous history of syncope, presyncope, cardiac arrest or documented ventricular arrhythmia. His regular medication included aspirin, bisoprolol, metformin, dapagliflozin, ezetimibe, lansoprazole and mirtazapine. High-sensitivity troponin was mildly elevated from 30 ng/L to 40 ng/L. His admission ECG demonstrated coved ST-segment elevation with T-wave inversion in V1-V2, consistent with a type 1 Brugada ECG pattern (Figure 1). Subsequent ECGs demonstrated evolving anterior ischaemic changes, prompting urgent coronary angiography.

**Figure 1:**
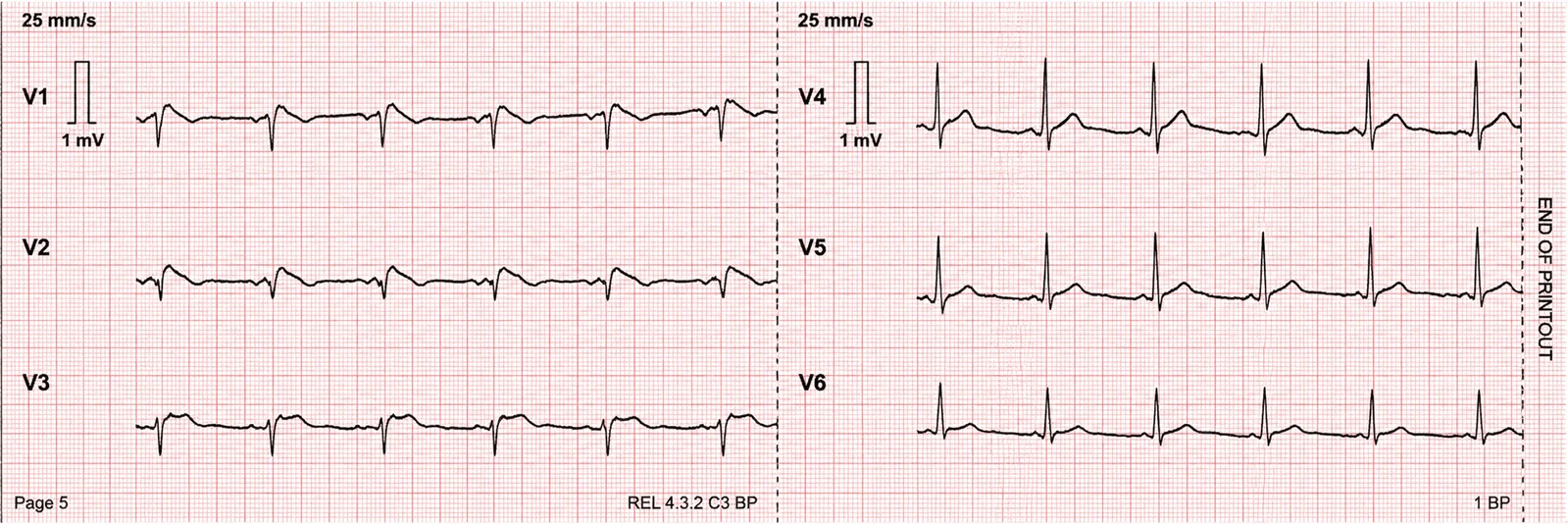
Twelve-lead ECG on admission demonstrating coved ST-segment elevation with T-wave inversion in V1–V2, consistent with a type 1 Brugada ECG pattern.

Coronary angiography demonstrated extensive multivessel coronary artery disease. There was moderate distal left main stem disease, chronic occlusion of the mid-left anterior descending artery with collateral filling, moderate circumflex disease, severe distal atrioventricular circumflex disease, and severe disease involving the mid-right coronary artery and posterior descending artery (Figures 2-4).

**Figure 2:**
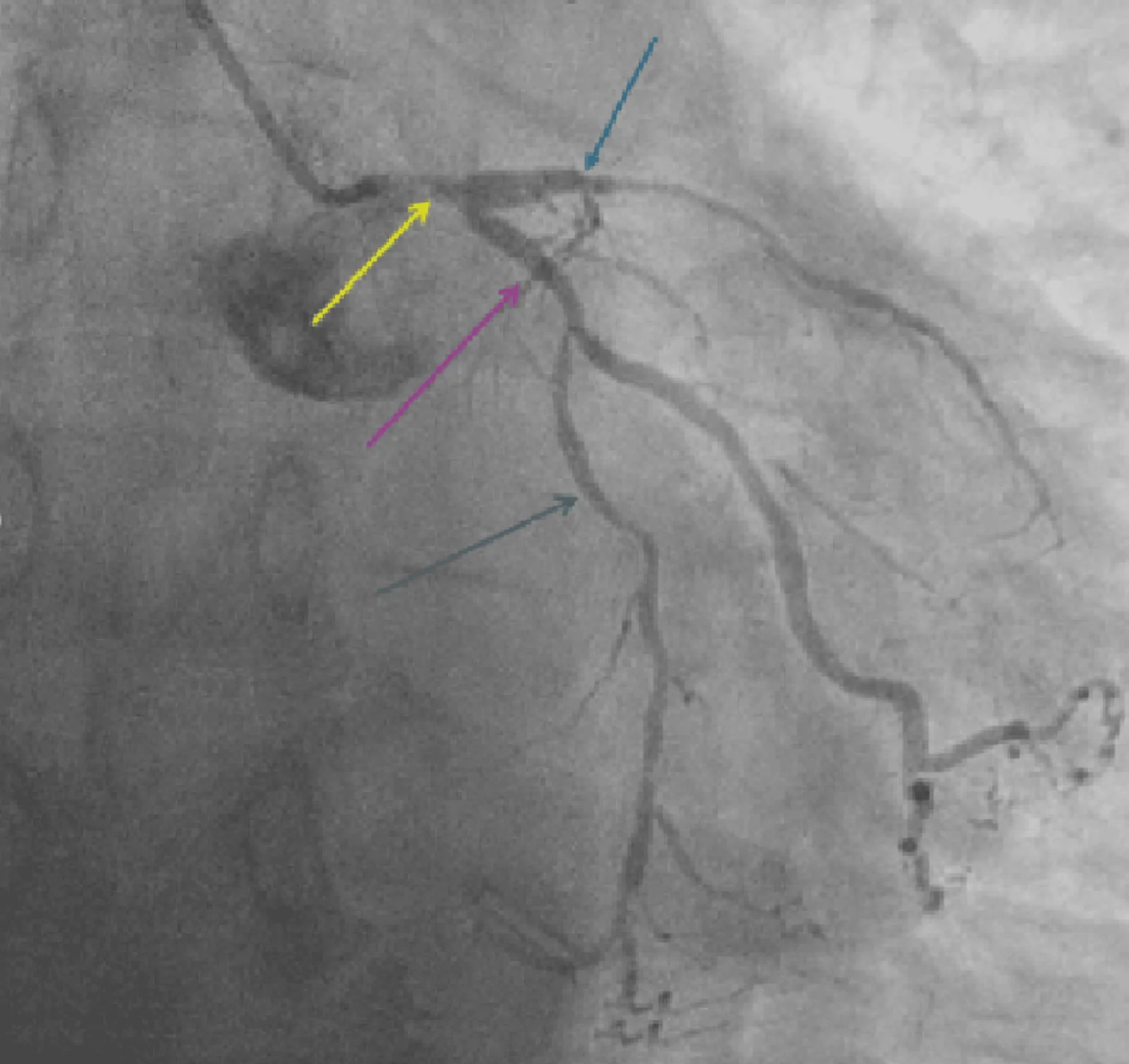
Posterior-anterior (PA) caudal view of coronary angiogram showing moderate distal left main stem disease. Left anterior descending artery (LAD) was occluded at the mid vessel with filling from the right coronary artery (RCA). Left circumflex artery (LCx) had moderate disease in the mid vessel with severe disease in the AV circumflex artery. Coronary angiography demonstrating multivessel disease. Yellow arrow showing distal left main stem disease. Blue arrow showing occluded LAD. Purple arrow showing circumflex artery. Green arrow showing atrioventricular circumflex artery.

**Figure 3:**
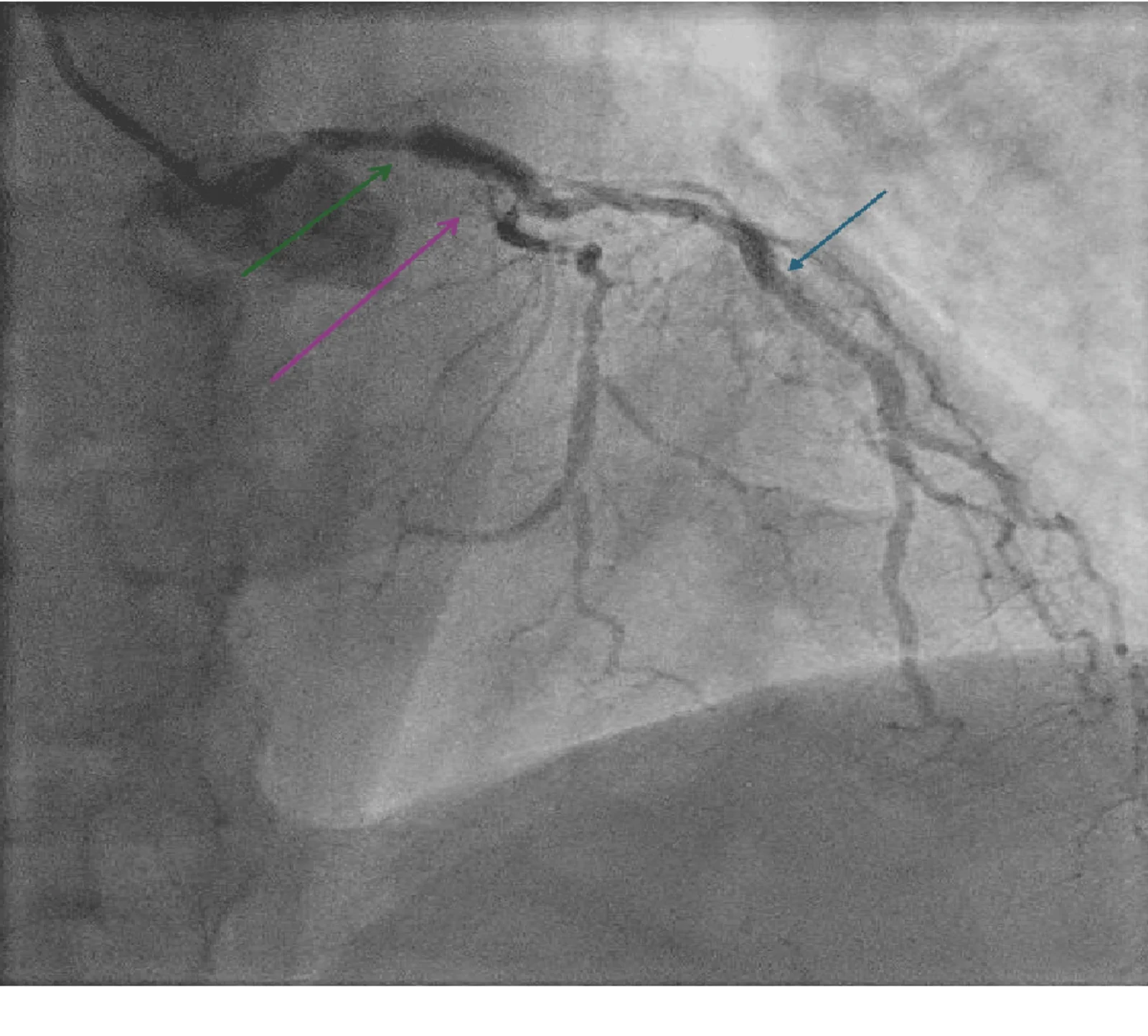
Left anterior oblique (LAO) cranial view of the left coronary artery showing occluded mid LAD with moderate distal left main stem disease. Green arrow demonstrating distal left main stem disease. Blue arrow showing circumflex artery. Purple arrow showing occluded LAD.

**Figure 4:**
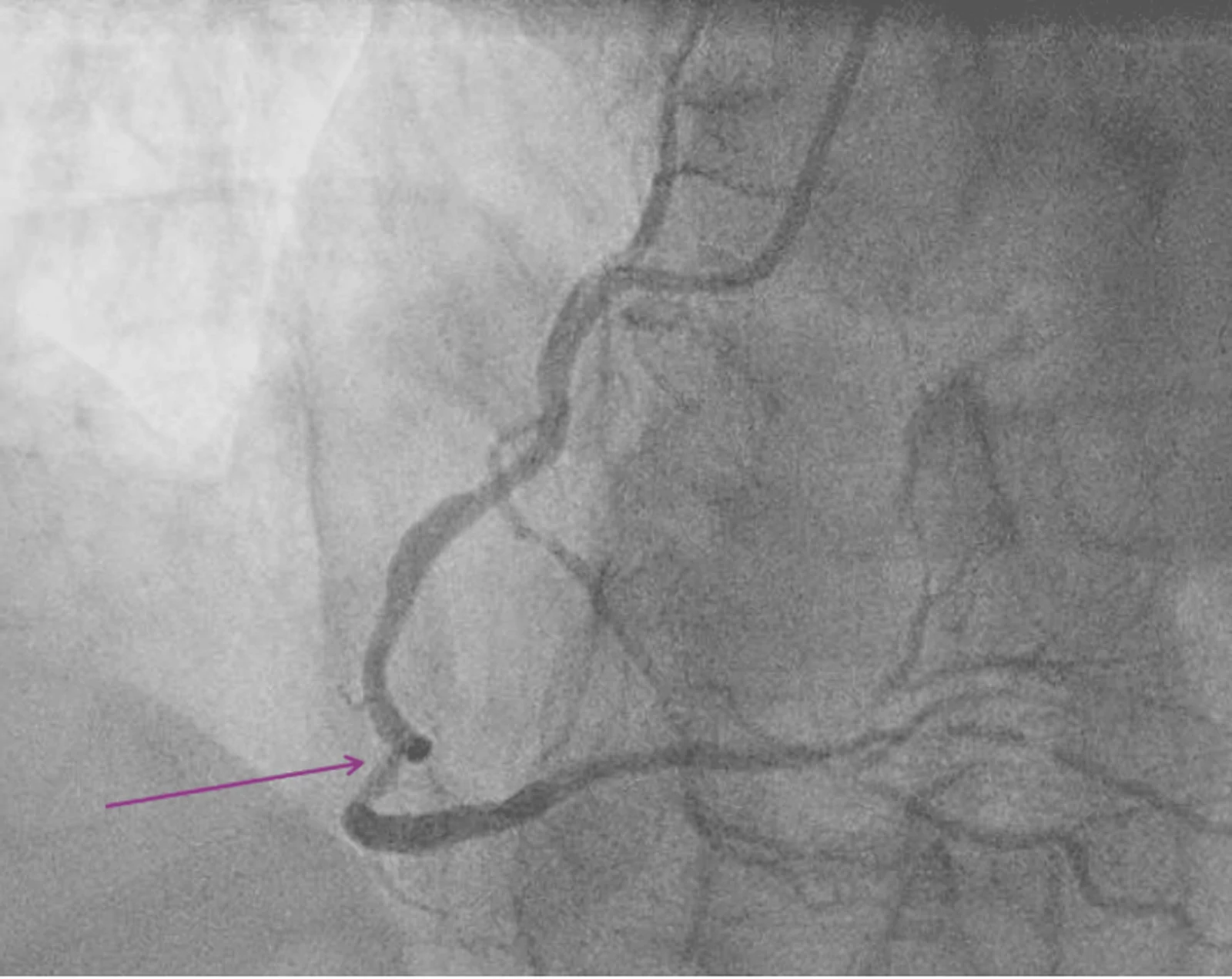
Left anterior oblique (LAO) cranial view of the right coronary artery (RCA). Purple arrow showing moderate to severe disease in mid-distal RCA.

The case was discussed by the Heart Team and surgical revascularisation was recommended. The patient underwent coronary artery bypass grafting with a left internal mammary artery graft to the left anterior descending artery and a saphenous vein graft to the obtuse marginal artery. The posterior descending artery was considered too small for grafting, and the distal right coronary artery was diffusely calcified and unsuitable for bypass. His postoperative recovery was uncomplicated. Serial ECGs following surgery showed complete resolution of the type 1 Brugada pattern (Figure 5).

**Figure 5:**
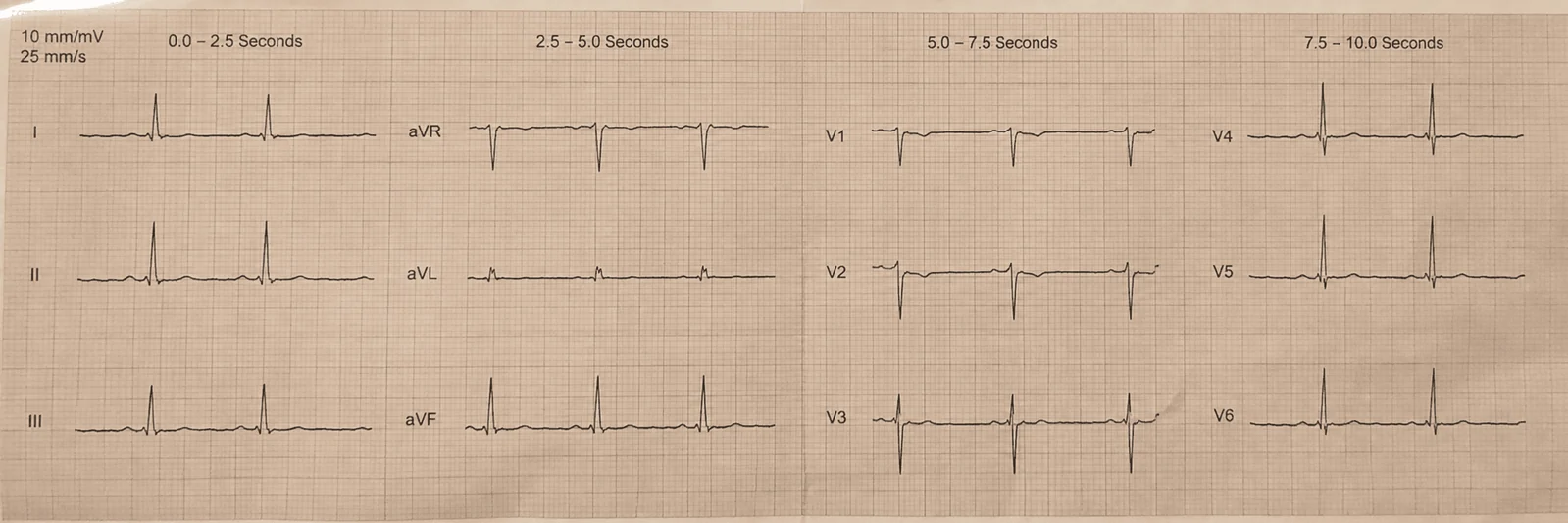
Twelve-lead ECG following coronary artery bypass grafting demonstrating complete resolution of the previous type 1 Brugada ECG pattern.

His regular medication, including mirtazapine, was continued throughout admission and following surgery. Following revascularisation, sodium-channel blocker provocation testing was not pursued in view of the absence of arrhythmic symptoms, documented ventricular arrhythmia or a family history of sudden cardiac death, together with complete resolution of the type 1 ECG pattern. The patient remained clinically well at outpatient follow-up. There was no recurrence of the type 1 Brugada ECG pattern and no reported syncope or arrhythmic symptoms.

## Discussion

Brugada syndrome is an inherited cardiac channelopathy associated with an increased risk of ventricular arrhythmia and sudden cardiac death. A type 1 Brugada ECG pattern may occur spontaneously or following sodium-channel blocker provocation. Similar ECG appearances have also been described in a number of reversible clinical conditions, including myocardial ischaemia, electrolyte disturbance, fever and drug exposure [1].

In this case, a type 1 Brugada ECG pattern was seen in a patient presenting with crescendo angina and a mild troponin rise. Subsequent ECGs showed evolving anterior ischaemic changes and coronary angiography demonstrated extensive multivessel coronary artery disease. Following surgical revascularisation, the type 1 pattern completely resolved.

This case adds to the limited literature describing transient type 1 Brugada ECG patterns associated with myocardial ischaemia. Xu et al. identified 17 published cases of myocardial ischaemia-associated Brugada phenocopy in the International Registry on Brugada Phenocopy [1]. In most cases, the ECG changes resolved following treatment of the underlying coronary pathology. Casu et al. reported a patient with unstable angina and severe three-vessel coronary artery disease in whom a type 1 Brugada ECG pattern resolved following coronary revascularisation [2]. Valdivieso et al. described transient Brugada ECG changes associated with myocardial ischaemia secondary to coronary steal, which also resolved following treatment [3]. Our case is distinguished by the dynamic electrocardiographic evolution from a type 1 Brugada pattern to overt ischaemic changes in the setting of extensive multivessel coronary disease requiring surgical revascularisation, followed by complete resolution of the Brugada phenotype despite continuation of mirtazapine. In the absence of arrhythmic syncope, documented ventricular arrhythmia or a family history of sudden cardiac death, these observations further support myocardial ischaemia as a plausible trigger for the transient ECG phenotype, while acknowledging that latent Brugada syndrome cannot be definitively excluded in the absence of pharmacological provocation testing.

The mechanism by which myocardial ischaemia produces a Brugada-like ECG pattern is not fully understood. Ischaemia can alter transmembrane ionic currents and produce regional differences in repolarisation, particularly within the right ventricular outflow tract. These changes may reproduce an ECG appearance similar to that seen in Brugada syndrome [1]. Resolution of these electrophysiological changes following restoration of myocardial perfusion provides a plausible explanation for the transient nature of the ECG findings observed in reported cases.

A pharmacologically mediated Brugada phenotype was also considered given concomitant mirtazapine therapy. However, mirtazapine was continued throughout the index admission and following surgical revascularisation, while the type 1 ECG pattern subsequently resolved. The absence of a temporal relationship between drug withdrawal and ECG normalisation therefore makes a drug-mediated phenotype less likely.

This case highlights the importance of considering myocardial ischaemia when a type 1 Brugada ECG pattern is identified in a patient presenting with symptoms suggestive of an acute coronary syndrome. The ECG should be interpreted in the context of the clinical presentation, and serial ECGs may be particularly useful in demonstrating the evolution of the underlying process.

## Conclusions

This case highlights the importance of recognising that a type 1 Brugada ECG pattern may occur in association with reversible clinical conditions, including myocardial ischaemia, and does not necessarily establish a diagnosis of inherited Brugada syndrome. Careful assessment of the clinical context, arrhythmic history, family history, and potential reversible precipitants is therefore essential when evaluating such patients.

The resolution of the type 1 Brugada pattern following coronary revascularisation in this patient supports myocardial ischaemia as a potential precipitating factor. However, resolution of the ECG abnormality after treatment of the suspected trigger does not completely exclude an underlying predisposition to Brugada syndrome. This case therefore emphasises the value of serial ECG assessment and cautious interpretation of transient Brugada patterns, particularly in patients presenting with significant coronary artery disease.
